# Supplementary material for: Intranasal mask for protecting the respiratory tract against viral aerosols
Source: Nat Commun. 2023 Dec 18;14:8398. doi: 10.1038/s41467-023-44134-w (PMC10728126; doi:10.1038/s41467-023-44134-w)
Supplement: Supplementary file 1 — Supplementary Information [file 41467_2023_44134_MOESM1_ESM.pdf]

Supplementary information for

Intranasal mask for protecting the respiratory tract against viral aerosols

Authors: Xiaoming Hu[1,2&], Shuang Wang[1,2&], Shaotong Fu[2,3&], Meng Qin[4], Chengliang Lyu[1], Zhaowen Ding[1], Yan Wang[1,2], Yishu Wang[1,2], Dongshu Wang[5], Li Zhu[5], Tao Jiang[6], Jing Sun[7], Hui Ding[8], Jie Wu[1,2], Lingqian Chang[9], Yimin Cui[10,11], Xiaocong Pang[10,11], Youchun Wang[12], Weijin Huang[12], Peidong Yang[13], Limin Wang[2,3*], Guanghui Ma[1,2*], Wei Wei[1,2*]

*Corresponding authors: weiwei@ipe.ac.cn; ghma@ipe.ac.cn; lmwang@ipe.ac.cn

The PDF file includes:

Figs. S1 to S36

References (1)

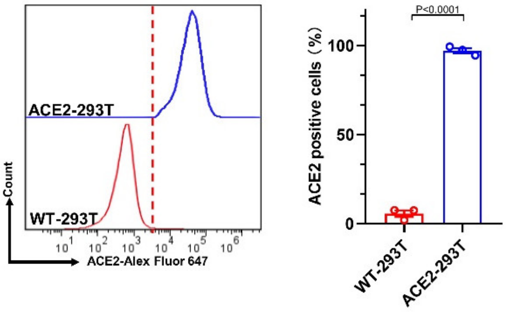

Fig. S1. Flow cytometry analysis of ACE2 protein expression on WT-293T and ACE2-293T cells, showing ACE2 protein overexpressed in ACE2-293T cells (right, n=3 biologically independent experiments). Data represent as means ± S.E.M. Statistical significance was calculated using two-tailed unpaired t-test. The P value of WT-293T to ACE2-293T was <0.0001. The experiment was repeated three times independently with similar results. Source data are provided in the Source data file.

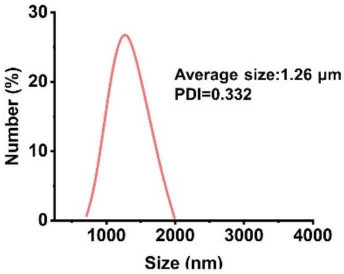

Fig. S2. Size distribution and corresponding particle dispersion index (PDI) of aMV in PBS solution, showing the average size of aMV was 1.26 μm (PDI=0.332). The experiment was repeated three times independently with similar results. Source data are provided in the Source data file.

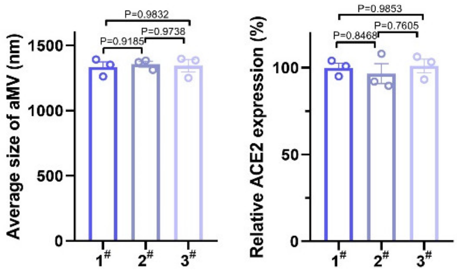

Fig. S3. Average size (left) and ACE2 expression level (right) of aMV in three different batches (n=3 biologically independent experiments). Data represent as means ± S.E.M. Statistical significance was calculated using one-way ANOVA with multiple comparison tests. All significant P-values were indicated. Source data are provided in the Source data file.

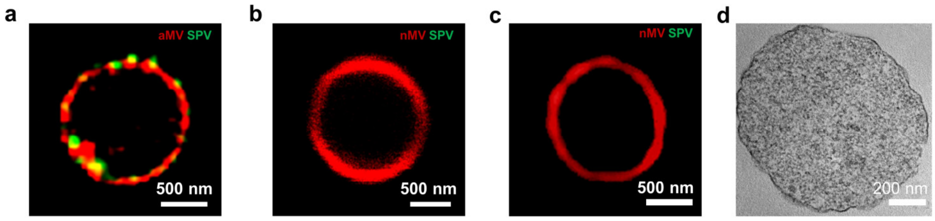

Fig. S4. STED and TEM images of MV interacted with SPV.

(a) STED image of aMV incubated with SPV for 30 minutes. The membrane of aMV was labeled with DiD (red), and SPV (wild-type) was labeled with FITC (green).

(b) STED image of nMV incubated with SPV for 30 minutes. The membrane of nMV was labeled with DiD (red), and SPV (wild-type) was labeled with FITC (green).

(c) STED image of nMV incubated with SPV for 3 h. The membrane of nMV was labeled with DiD (red), and SPV (wild-type) was labeled with FITC (green).

(d) TEM image of nMV incubated with SPV for 3 h.

The result showed the SPV signal neither on the surface of nMV nor within the nMV, thus excluding nonspecific binding of SPV on MV. The experiments were repeated three times independently with similar results.

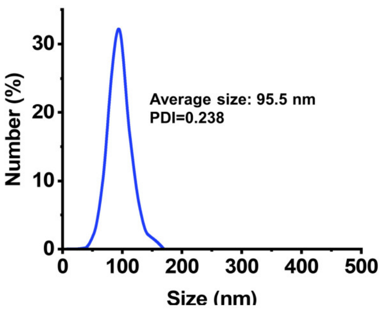

Fig. S5. Size distribution and corresponding PDI of aNV in PBS solution, showing the average size of aNV was 95.5 nm (PDI=0.238). The experiment was repeated three times independently with similar results. Source data are provided in the Source data file.

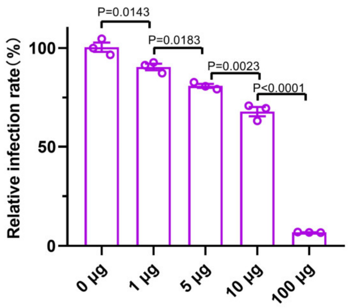

Fig. S6. Relative infection rates of ACE2-293T cells that were treated with different doses of aMV (0, 1, 5, 10, and 100 μg) and then challenged with SPV (wild-type) (n=3 biologically independent experiments). The relative infection rates decreased with the increase of aMV dose, indicating a dose-dependent protective effect. Data represent as means ± S.E.M. Statistical significance was calculated using one-way ANOVA with multiple comparison tests. All significant P-values are indicated. Source data are provided in the Source data file. The experiment was repeated three times independently with similar results.

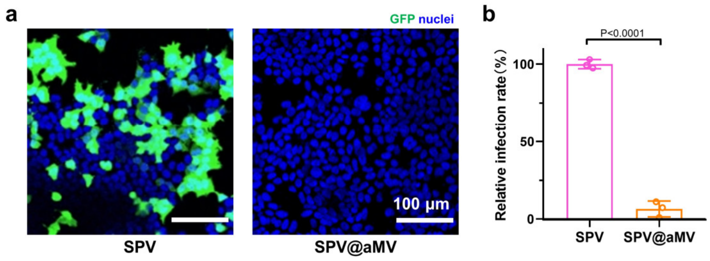

Fig. S7. Infection ability of SPV that were entrapped by aMV. After treating SPV (wild-type) with 100 μg aMV for 24 h, we collected the SPV that had been entrapped by aMV (SPV@aMV) and added them to ACE2-293T cells culture. Meanwhile, the same dose of SPV was also added to ACE2-293T cells as the positive control.

(a) CLSM images of ACE2-293T cells that were challenged with SPV or SPV@aMV. The infected cells expressed GFP protein (green), and the nuclei were stained with DPAI (blue).

(b) Relative infection rates of ACE2-293T cells that were challenged with SPV or SPV@aMV (n=3 biologically independent experiments).

Data in b represent as means ± S.E.M. Statistical significance was calculated using two-tailed unpaired t-test. The P-value of SPV to SPV@aMV was < 0.0001. The experiment was repeated three times independently with similar results. Source data are provided in the Source data file.

Compared with SPV, SPV@aMV showed significantly reduced infection rate to ACE2-293T cells. CLSM image showed that no GFP signal existed in SPV@aMV challenged cells, indicating that SPV lost the ability to infect ACE2-293T cells after they were entrapped by aMV.

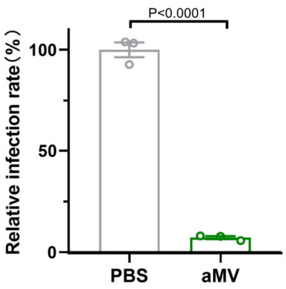

Fig. S8. Relative infection rate of ACE2-293T cells that were treated with PBS or 100 μg aMV that had been stored in sterile PBS at 4 °C for 3 months, and then challenged with SPV (wild-type) (n=3 biologically independent experiments). Similar to the freshly prepared aMV, aMV after above long-term storage achieved potent protective effect by reducing the viral infection rate to 7.2 %, indicating the good long-term storage stability of aMV. Data represent as means ± S.E.M. Statistical significance was calculated using two-tailed unpaired t-test. The P-value of PBS to aMV was < 0.0001. The experiment was repeated three times independently with similar results. Source data are provided in the Source data file.

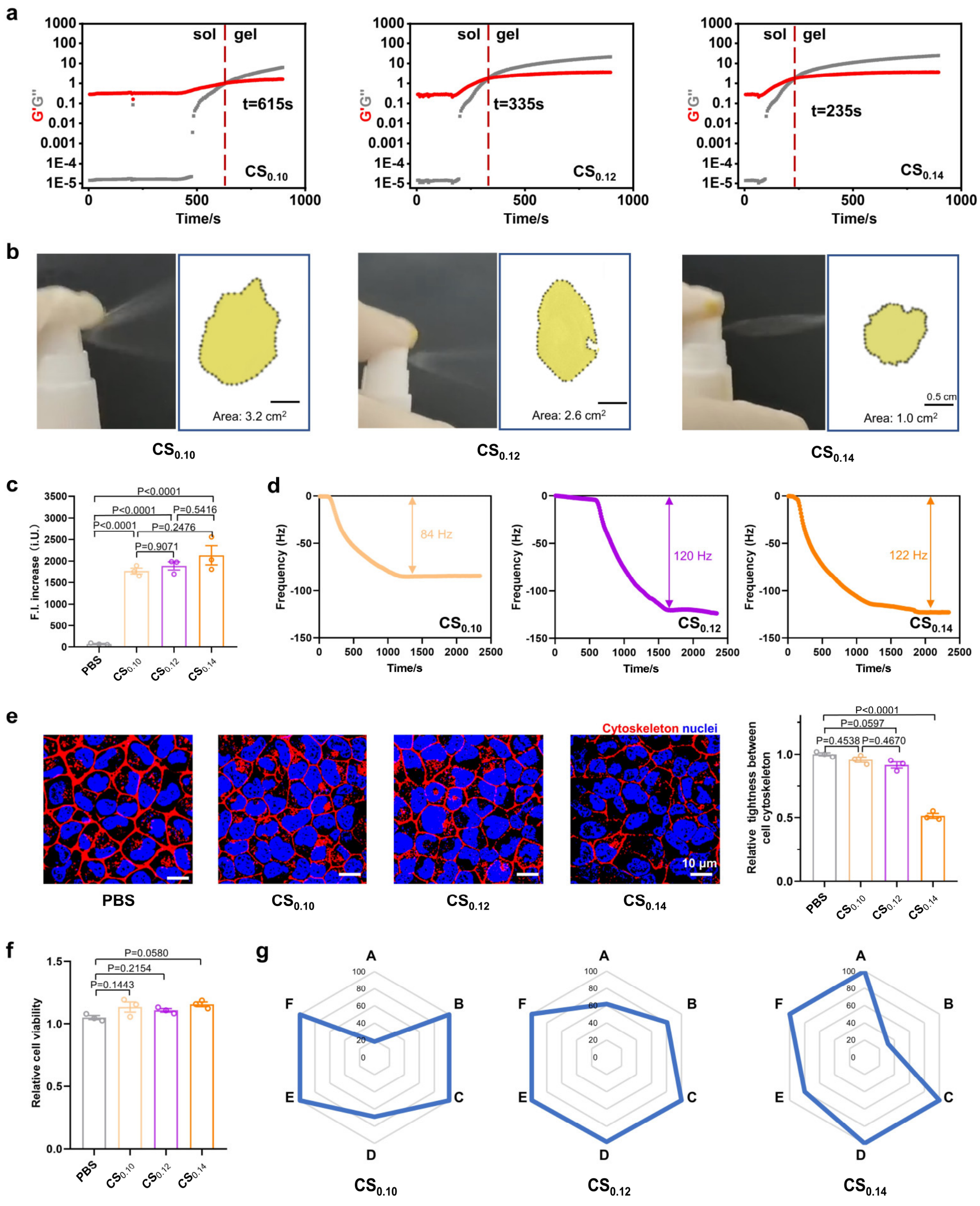

Fig. S9. Screening of ideal hydrogel with appropriate properties, including thermosensitive gelling property, spray characteristic, interception ability to SPV aerosols, interaction force with SPV, effect on cell tight junction, and cytotoxicity.

(a) The evolution of dynamic loss modulus (G′, red) and storage modulus (G″, gray) of $CS_{0.10}$ (10 mg/ml chitosan with 200 mg/ml β-sodium glycerophosphate), $CS_{0.12}$ (12 mg/ml with 200 mg/ml β-sodium glycerophosphate) and $CS_{0.14}$ (14 mg/ml with 200 mg/ml β-sodium glycerophosphate) at 37 °C. When G″>G′, the hydrogel transformed from the liquid state to the gel state.

(b) Photographic images showing the spray characteristic of $CS_{0.10}$, $CS_{0.12}$, and $CS_{0.14}$ (left), accompanied with the corresponding spray area (right) formed on the paper 10 cm away from the sprayer.

(c) The fluorescence intensity (F.I.) increase of $CS_{0.10}$, $CS_{0.12}$, and $CS_{0.14}$ after the interception of

Cy5-stained viral aerosols (n=3 biologically independent experiments).

(d) Quartz crystal microbalance curve of $CS_{0.10}$, $CS_{0.12}$, and $CS_{0.14}$ during interaction with SPV. The downward frequency reflected an increased mass on the chip, thus reflecting the interaction force between hydrogel and SPV.

(e) CLSM images of human nasal epithelial cells after incubation with $CS_{0.10}$, $CS_{0.12}$, or $CS_{0.14}$ for 1 h, accompanied with the corresponding quantitative data of relative cell tight junction degree (n=3 biologically independent experiments). The cytoskeleton was stained with FITC-phalloidin (red, false color), and cell nuclei were stained with DAPI (blue). The cytoskeleton exhibited signs of discontinuity or even disappearance when the chitosan concentration increased to $CS_{0.14}$, indicating the reduced and damaged tight connections between the nasal epithelial cells.

(f) Relative cell viability of human nasal epithelial cells after incubation with $CS_{0.10}$, $CS_{0.12}$, or $CS_{0.14}$ for 1 h (n=3 biologically independent experiments).

(g) Radar images of $CS_{0.10}$, $CS_{0.12}$, and $CS_{0.14}$ showing the indicated parameters. A: relative thermosensitive gelling property; B: relative spray characteristic; C: relative interception ability to SPV aerosols; D: relative interaction force with SPV; E: relative effect on cell tight junction; F: relative cytotoxicity. To generate the radar images, the highest value of each parameter was normalized as a score of 100, other values in the relative parameter were then calculated as the corresponding scores. After normalizing these six aspects, the area (enclosed by the blue line) of these three recipes in the radar map was calculated. Considering the overall performance in the above six aspects, we chose $CS_{0.12}$ as our final hydrogel recipe.

Data in c, e, and f represent as means ± S.E.M. Statistical significance in c, e, and f were calculated using one-way ANOVA with multiple comparison tests. The experiment was repeated three times independently with similar results. Source data are provided in the Source data file.

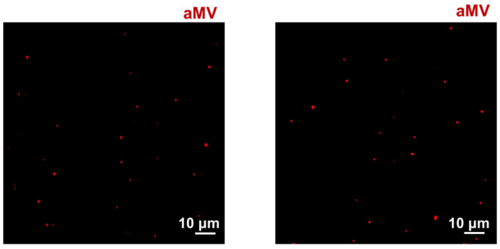

Fig. S10. CLSM images of aMV@GEL upon initial mixture of aMV and GEL (left) and upon redispersion after 24 h storage in refrigerator (right). The aMV was initially mixed with liquid hydrogel by shaking 10 times. The liquid aMV@GEL was then stored in the refrigerator for 24 hours, followed by shaking for 10 times to redisperse the aMV in the liquid GEL. The aMV was stained by DiD (red). The experiment was repeated three times independently with similar results.

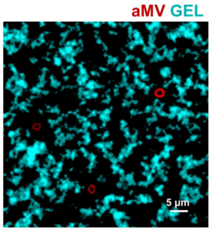

Fig. S11. CLSM image showing the aMV's vesicle structure in hydrogel at 24 h post aMV@GEL gelation. The aMV was stained by DiD (red), the GEL was stained by FITC (cyan, false color). The experiment was repeated three times independently with similar results.

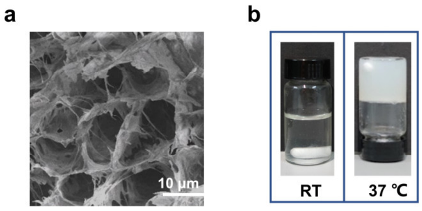

Fig. S12. Porous structure and thermosensitive gelling characteristic of $CS_{0.12}$.

(a) SEM image of $CS_{0.12}$ showing the porous structure with pore size of 10-20 μm.

(b) Photographic images showing $CS_{0.12}$ with a liquid state at RT while a gel state at 37 °C.

The experiments were repeated three times independently with similar results. Source data are provided in the Source data file.

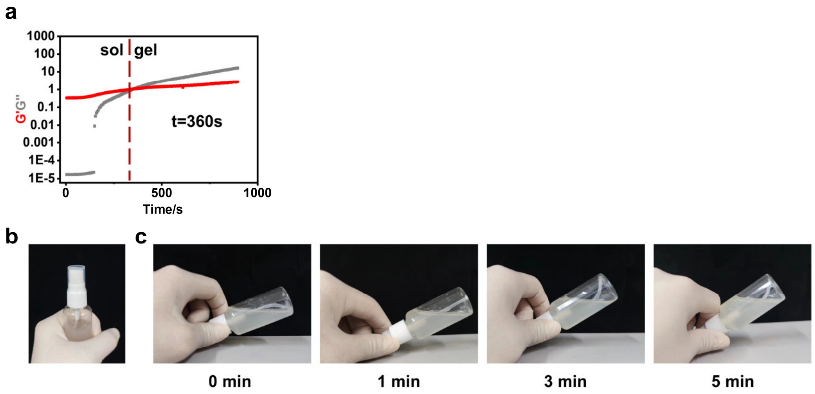

Fig. S13. Thermosensitivity of aMV@GEL.

(a) Evolution of dynamic loss modulus (G′, red) and storage modulus (G″, gray) of aMV@GEL at 37°C. When G″>G′, aMV@GEL transformed from liquid state to gel state.

(b) Photographic image of aMV@GEL that was taken out from the 4°C refrigerator and was held with warm hand.

(c) Photographic images of aMV@GEL that was held with warm hand for the indicated times.

The experiments were repeated three times independently with similar results. Source data are provided in the Source data file.

The aMV@GEL maintained liquid state even at 5 min, thus providing sufficient time window for the spray into the nasal cavity.

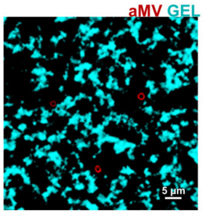

Fig. S14. CLSM image showing the aMV remained vesicle structure in hydrogel after experiencing spray-induced shear rates. aMV were labeled with DiD (red), and the GEL was stained with FITC (cyan, false color). The experiment was repeated three times independently with similar results.

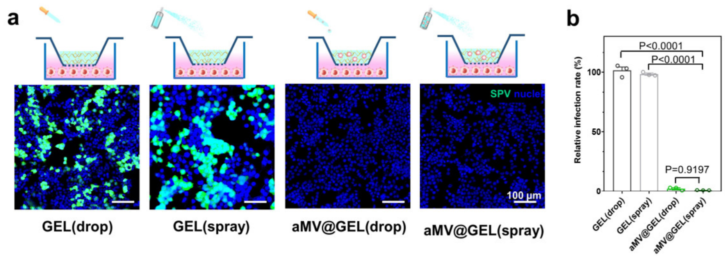

Fig. S15. Protection effect of aMV@GEL after experiencing spray-induced shear rates.

(a) Schematic illustration of a Transwell™ model for evaluating the infection rate of SPV (wild-type) challenged ACE2-293T cells (in the bottom chamber) with GEL (drop), GEL (spray), aMV@GEL (drop) or aMV@GEL (spray) treatment (in upper chamber) and the corresponding representative CLSM images of ACE2-293T cells. The infected cells expressed GFP protein (green), and the nuclei were stained with DPAI (blue).

(b) Relative infection rates of ACE2-293T cells in the GEL (drop), GEL (spray), aMV@GEL (drop) and aMV@GEL (spray) groups (n=3 biologically independent experiments).

Data represent as means ± S.E.M. Statistical significance was calculated using one-way ANOVA with multiple comparison tests. All significant P-values are indicated. The experiments were repeated three times independently with similar results. Source data are provided in the Source data file.

Compared with the obvious SPV infection (green) in the GEL (drop) and GEL (spray) groups, a substantially reduced SPV infection was observed in both the aMV@GEL (drop) and aMV@GEL (spray) groups. Note that the reduction showed no significance between aMV@GEL (drop) and aMV@GEL (spray) groups, indicating that the aMV in the GEL retained their antiviral efficacy even after experiencing spray-induced shear rates.

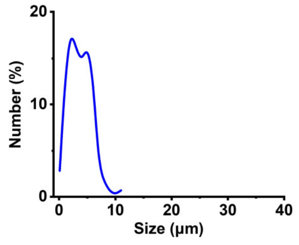

Fig. S16. Size distribution of aerosols produced by the aerosol generator. The vast majority of viral aerosols ranged from 1 μm to 10 μm, which was the same as real viral aerosols[1]. The experiment was repeated two times independently with similar results. Source data are provided in the Source data file.

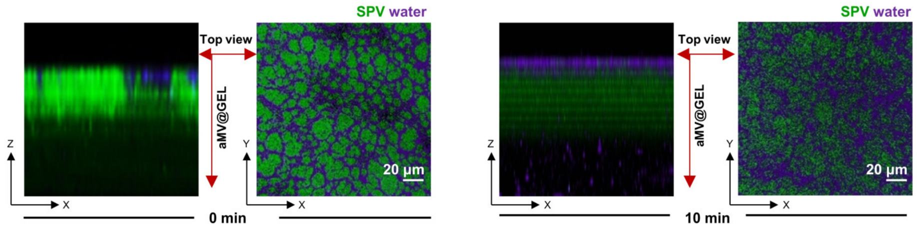

Fig. S17. CLSM images of SPV aerosols after nebulizing to aMV@GEL for 0 min (left, contacting) and 10 min (right, fusing). The x-z images showed that the SPV aerosols released the SPV into the downward inferior aMV@GEL. We dissolved fluorescein sodium in the water of SPV aerosols (purple, false color), and the SPV in SPV aerosols were stained with Cy5 (green, false color). The experiment was repeated two times independently with similar results.

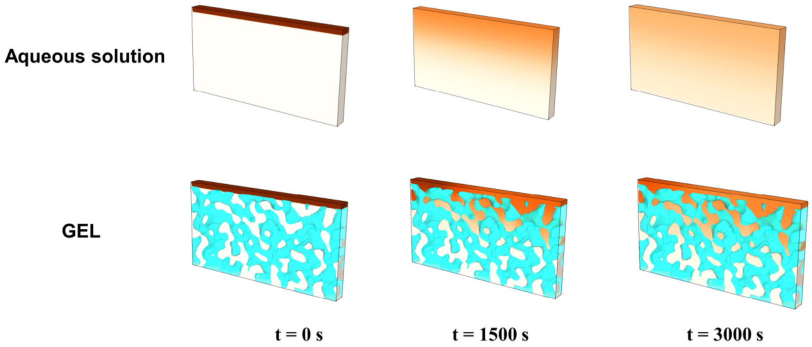

Fig. S18. Simulation depicting the diffusion of the virus in aqueous solution or GEL. The aqueous solution was represented by the white color, virus was represented by the orange color, and the GEL framework was represented by the cyanine color. The virus exhibited a slower movement towards the interior of the GEL compared to that of aqueous solution. Such a reduced diffusion of virus within the GEL facilitated the encounter with the MV, ensuring the efficient capture and entrapment of the virus by the MV. The experiment was repeated three times independently with similar results.

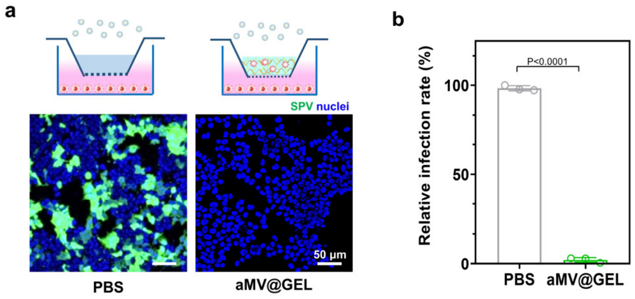

Fig. S19. aMV@GEL protected ACE2-293T cells against SPV aerosol infection *in vitro*.

(a) Schematic illustration of a Transwell™ model for evaluating the infection rate of SPV (wild-type) challenged ACE2-293T cells (in the bottom chamber) with PBS or aMV@GEL treatment (in upper chamber) and the corresponding representative CLSM images of ACE2-293T cells. The SPV aerosols were nebulized over the Transwell™ model. The infected cells expressed GFP protein (green), and the nuclei were stained with DPAI (blue).

(b) Corresponding quantitative data in a (n=3 biologically independent experiments), indicating aMV@GEL provided an adequate protective effect for the ACE2-293T cells against SPV aerosols. Data in b represent as means ± S.E.M. Statistical significance in b was calculated using two-tailed unpaired t-test. The P-value of PBS to aMV@GEL was <0.0001. The experiment was repeated three times independently with similar results. Source data are provided in the Source data file.

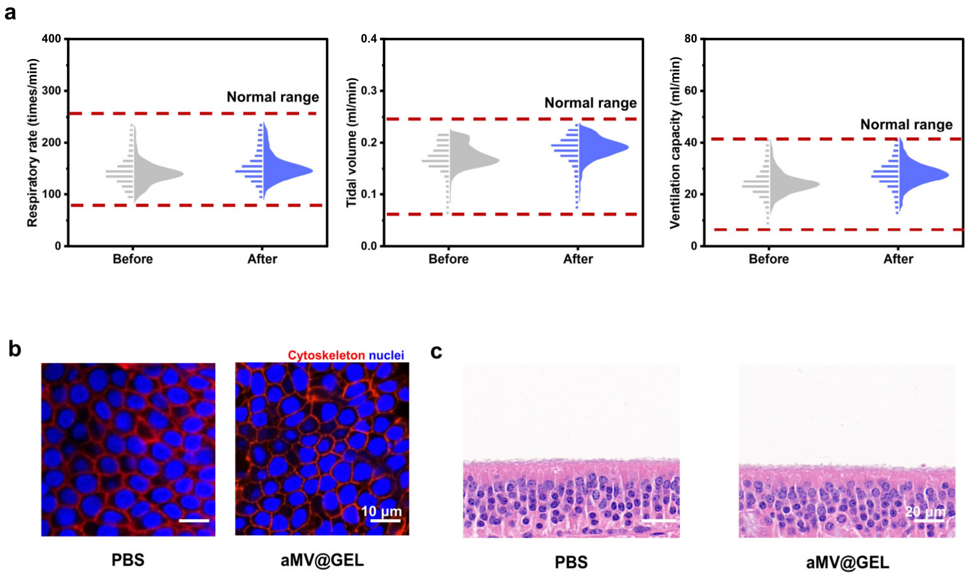

Fig. S20. Biosafety evaluations of aMV@GEL in mice

(a) The respiratory rate, tidal volume and ventilation capacity of mice before and after intranasal administration with aMV@GEL. The above three indicators were all in the normal ranges, indicating the existence of aMV@GEL did not disturb the normal breathing of the mice.

(b) Representative CLSM images of mice nasal mucosa at 1 h post intranasal administration with PBS or aMV@GEL, indicating aMV@GEL did not destroy the cytoskeleton. The cytoskeleton was stained with FITC-phalloidin (red, false color), and the nuclei were stained with DAPI (blue).

(c) Representative H&E images of the nasal mucosa at 1 h post intranasal administration with PBS or aMV@GEL.

The experiments were repeated three times independently with similar results. Source data are provided in the Source data file.

The data showed that aMV@GEL did not cause obvious damage to the structure of nasal mucosa.

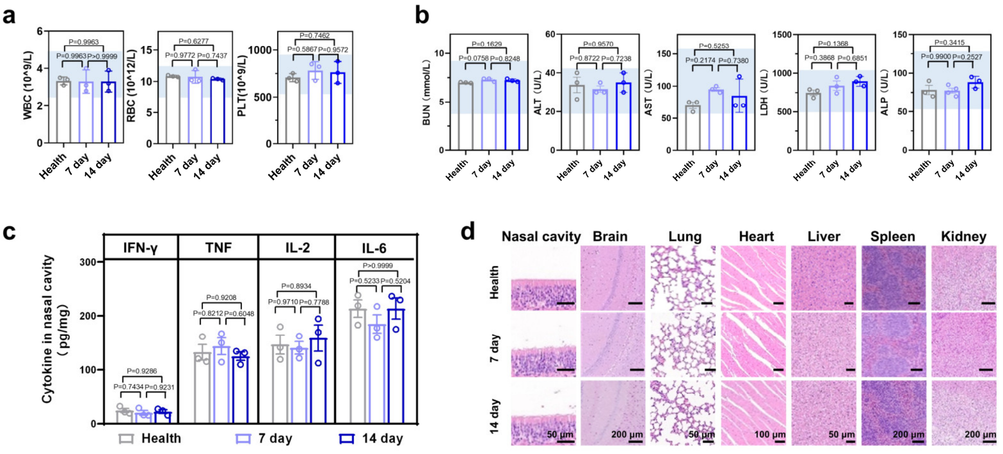

Fig. S21. Biosafety evaluations upon multiple aMV@GEL administrations. aMV@GEL was intranasally administered to mice every other day for a period of two weeks. At day 7 and day 14, the blood and the major tissue (including the nasal cavity, brain, lung, heart, liver, spleen, and kidney) were collected for biosafety evaluations.

(a) Blood analysis of white blood cells (WBC), red blood cell (RBC), and platelet (PLT) at day 7 and day 14 (n=3 biologically independent mice). The blue background presented the normal range.

(b) Serum biochemistry indices analysis of urea nitrogen (BUN), aspartate alanine aminotransferase (ALT), aminotransferase (AST), alkaline phosphatase (ALP), and lactate dehydrogenase (LDH) at day 7 and day 14 (n=3 biologically independent mice). The blue background presented the normal range.

(c) Inflammatory cytokines (IFN-γ, TNF, IL-2 and IL-6) analysis of the nasal homogenate at day 7 and day 14 (n=3 biologically independent mice).

(d) Representative H&E images of major tissues at day 7 and day 14.

Data in a, b and c represent as means ± S.E.M. Statistical significance in a, b and c were calculated using one-way ANOVA with multiple comparison tests. The significant P-values were indicated. The experiments were repeated two times independently with similar results. Source data are provided in the Source data file.

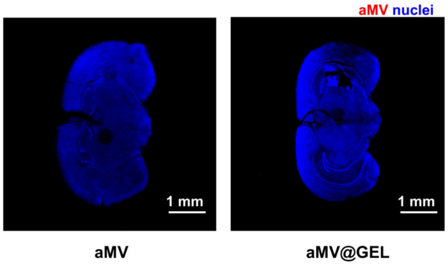

Fig. S22. Representative CLSM images of mouse brain at 8 h post intranasal administration with aMV or aMV@GEL. The aMV was stained with Cy5 (red) and the nuclei were stained with DAPI (blue). The data indicated that neither free aMV nor aMV@GEL transferred to the brain from the nasal cavity. The experiment was repeated three times independently with similar results.

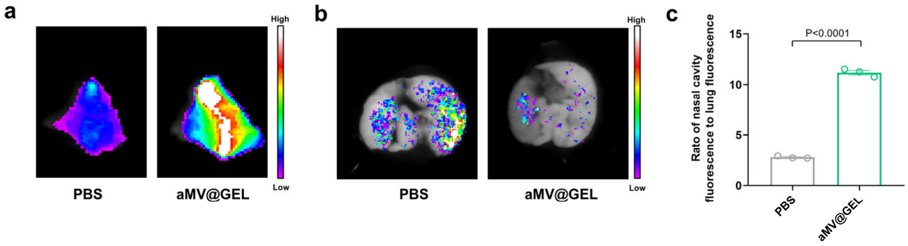

Fig. S23. Distribution of SPV in nasal cavity and lung of the mice with PBS or aMV@GEL intranasal administration.

(a) Representative e*x vivo* fluorescence images of mouse nasal cavity after 30 min inhalation of Cy5-SPV aerosols with or without aMV@GEL protection, indicating the aMV@GEL could intercept Cy5-SPV aerosols in the nasal cavity.

(b) Representative *ex vivo* fluorescence images of mouse lung after 30 min inhalation of Cy5-SPV aerosols with or without aMV@GEL protection, indicating the aMV@GEL could reduce the amount of Cy5-SPV in the lung.

(c) The corresponding ratio of the fluorescence in the mouse nasal cavity to that in the lung with or without aMV@GEL protection (n=3 biologically independent experiments). Compared with the unprotected PBS group, the above ratio significantly increased in the aMV@GEL group, emphasizing the viral aerosol interception effect of aMV@GEL in the nasal cavity and its lung protection effect.

Data in c represent as means ± S.E.M. Statistical significance in c was calculated using two-tailed unpaired t-test. The P value of PBS to aMV@GEL was <0.0001. The experiment was repeated two times independently with similar results. Source data are provided in the Source data file.

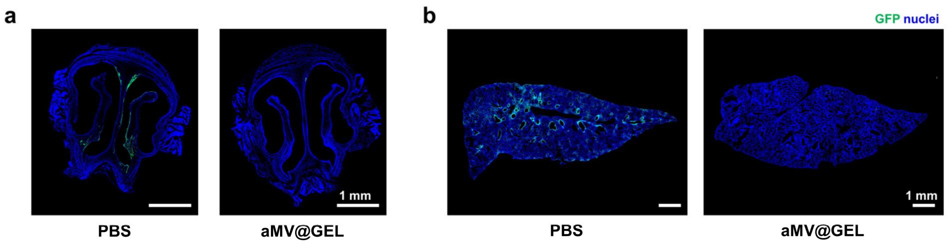

Fig. S24. Representative CLSM images of mouse nasal cavity (a) and lung (b) after 3 days of challenge with SPV variant (B.1.1.529, Omicron) aerosols. GFP was produced by SPV-infected cells (green), and the cell nuclei were stained with DAPI (blue). The data showed that aMV@GEL could protect the nasal cavity and lung of mice from the variant SPV aerosols. The experiment was repeated three times independently with similar results.

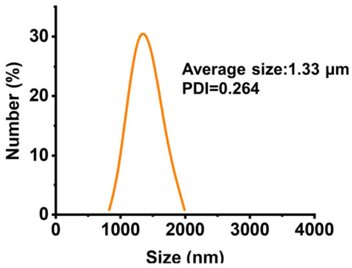

Fig. S25. Size distribution and the corresponding PDI of sMV in PBS solution, showing the average size of sMV was 1.33 μm (PDI=0.264). The experiment was repeated three times independently with similar results. Source data are provided in the Source data file.

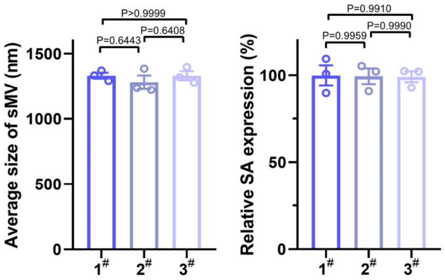

Fig. S26. Average size (left) and SA expression level (right) of sMV in three different batches. Data represent as means ± S.E.M. Statistical significance was calculated using one-way ANOVA with multiple comparison tests. Source data are provided in the Source data file.

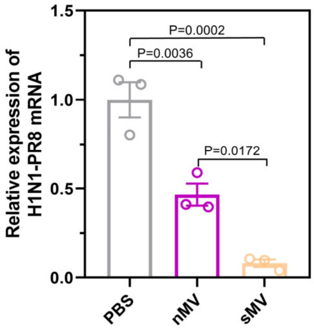

Fig. S27. Relative level of H1N1-PR8 mRNA in MDCK cells of different groups (PBS, 50 μg nMV, 50 μg sMV) (n=3 biologically independent experiments). Data represent as means ± S.E.M. Statistical significance was calculated using one-way ANOVA with multiple comparison tests. The P-values of PBS to nMV, PBS to sMV, nMV to sMV were 0.0036, 0.0002, and 0.0172, respectively. The experiment was repeated three times independently with similar results. Source data are provided in the Source data file.

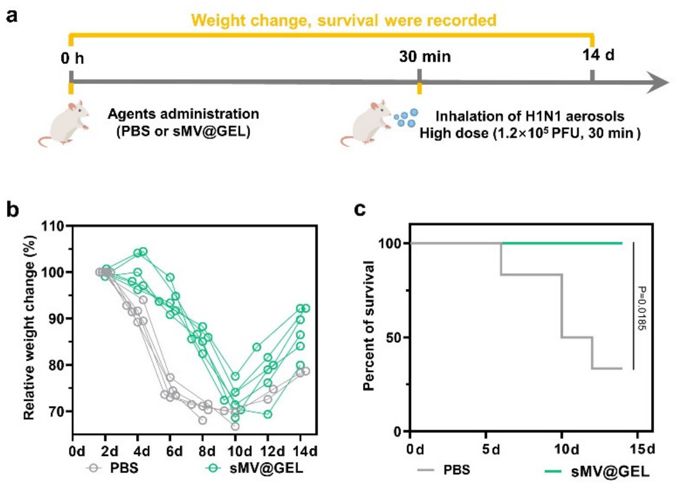

Fig. S28. Protection effect of sMV@GEL against high-dose H1N1-CA07 aerosols in mice.

(a) Schematic diagram for evaluating the *in vivo* protection effect of sMV@GEL against H1N1-CA07 aerosols in mice. On day 0, mice were given intranasal administration of PBS or sMV@GEL and were challenged with the indicated H1N1-CA07 viral aerosols. The weight change and survival status of mice with high dose challenge were recorded within 14 days.

(b) Weight change of mice in PBS and sMV@GEL groups within 14 days after being challenged with high dose H1N1-CA07 aerosols (n=6 biologically independent mice). One mouse in PBS group succumbed to the infection on day 6, two on day 10, and one on day 12. The weight data of these mice were excluded from the analysis at the corresponding days.

(c) Survival curves of mice in PBS and sMV@GEL groups within 14 days after being challenged with high dose H1N1-CA07 aerosols (n=6 biologically independent mice).

Statistical significance in c was tested with log-rank test. The P value of PBS to sMV@GEL was 0.0185. The experiment was repeated two times independently with similar results. Source data are provided in the Source data file.

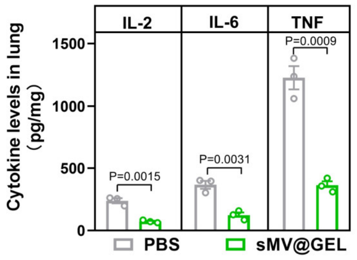

Fig. S29. The indicated inflammatory cytokines levels in the lung tissue of PBS and sMV@GEL treated mice after 7 days of challenge with H1N1-CA07 aerosols (n=3 biologically independent mice). Data represent as means ± S.E.M. Statistical significance was calculated using two-tailed unpaired t-test. All significant P-value are indicated. The experiment was repeated two times independently with similar results. Source data are provided in the Source data file.

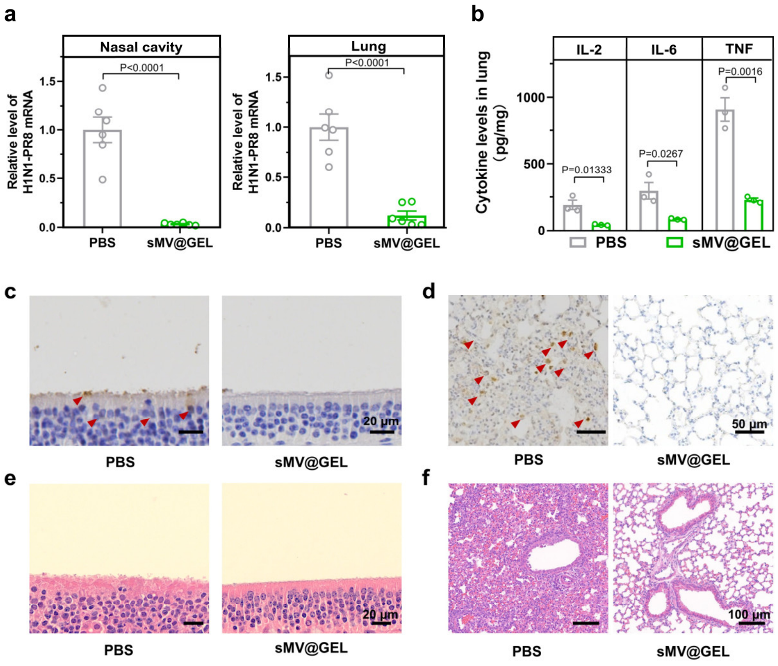

Fig. S30. *In vivo* protection effect of sMV@GEL to H1N1-PR8 aerosols.

(a) Relative level of H1N1-PR8 mRNA in the nasal cavity (left) and lung (right) in the PBS group and sMV@GEL group after 7 days of challenge with H1N1-PR8 aerosols (n=6 biologically independent mice).

(b) The indicated inflammatory cytokines levels in the lung tissue of the PBS group and sMV@GEL group after 7 days of challenge with H1N1-PR8 aerosols (n=3 biologically independent mice).

(c) Representative immunohistochemistry (IHC) sections of the nasal cavity in PBS group (left) and sMV@GEL group (right) after 7 days of challenge with H1N1-PR8 aerosols. The red arrows indicated the N protein of H1N1-PR8 (brown). The data showed that sMV@GEL provided strong protection to the nasal cavity of mice against H1N1-PR8 aerosols.

(d) Representative IHC sections of the lung in the PBS (left) and sMV@GEL (right) groups after 7 days challenged with H1N1-PR8 aerosols. The red arrows indicated the N protein of H1N1-PR8 (brown). The data showed that sMV@GEL provided strong protection to the lung of mice against H1N1-PR8 aerosols.

(e) Representative H&E sections of the nasal cavity in PBS group (left) and sMV@GEL group (right) after 7 days of challenge with H1N1-PR8 aerosols. The data showed that nasal mucosa epithelia was damaged in the PBS group, whereas the above phenomenon was not detected in the sMV@GEL treated mice.

(f) Representative H&E sections of the lung in the PBS (left) and sMV@GEL (right) groups after 7 days of challenge with H1N1-PR8 aerosols. The data showed that necrotic cells were positioned around the alveoli with obvious pulmonary inflammatory infiltration in the PBS group, whereas the

above phenomenon was not detected in the sMV@GEL treated mice.

Data in a and b represent as means ± S.E.M. Statistical significance in a and b were calculated using two-tailed unpaired t-test. All significant P-value are indicated. The experiment was repeated two times independently with similar results. Source data are provided in the Source data file.

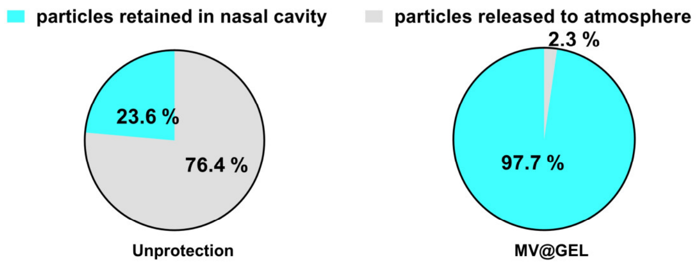

Fig. S31. Percentage of exhalant viral aerosols that were retained in the nasal cavity and released to the atmosphere under the unprotection state or MV@GEL protection state at 3 s (1.5 s inhalation followed by 1.5 s exhalation). The experiment was repeated three times independently with similar results.

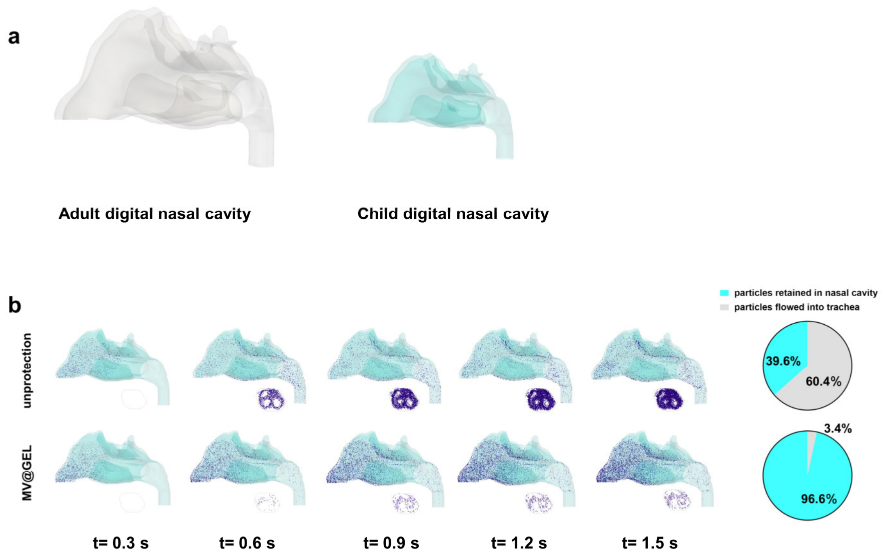

Fig. S32. Prediction of viral aerosol interception effect of MV@GEL in child digital nasal cavity by using CFD-DPM simulation.

(a) Comparation of 3D human adult and child digital nasal cavity model.

(b) Distribution of inhaled viral aerosols (blue dot) at different time points (0.3 s, 0.6 s, 0.9 s, 1.2 s, and 1.5 s) of unprotection situation (upper) or MV@GEL situation (lower) after the beginning of inhalation (left), and the corresponding percentage of viral aerosols that retained in the child nasal cavity or flowed into trachea at 1.5 s (right).

The experiment was repeated three times independently with similar results.

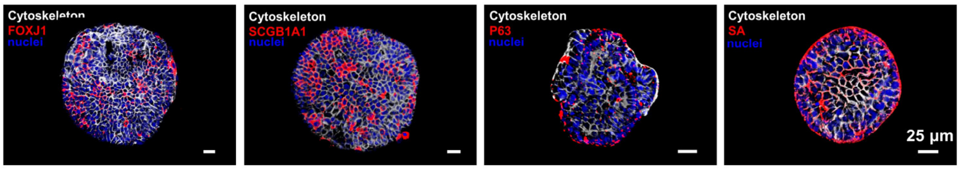

Fig. S33. Representative 3D reconstructed CLSM images showing expression of FOXJ1 (ciliated cell marker), SCGB1A1 (club cell marker), P63 (basal cell marker), and SA (H1N1 receptor) in lung organoids. FOXJ1, SCGB1A1, and P63 were stained with corresponding antibodies and secondary Alexa Fluor® 647 fluorescent antibodies (red), SA was stained with Cy3-SNL (red, false color), cytoskeleton was stained with FITC-phalloidine (white, false color), and cell nuclei were stained with DAPI (blue). The experiments were repeated two times independently with similar results.

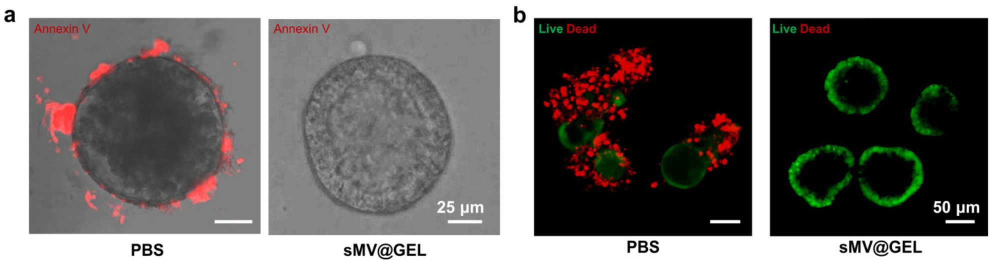

Fig. S34. Protection effect of sMV@GEL against H1N1-CA07 viral aerosol in HRT model.

(a) Cell apoptosis analysis of lung organoid in different groups after 48 h of challenge with H1N1-CA07 aerosols. The phosphatidylserine (red) was stained by Annexin V-mCherry.

(b) Live/Dead analysis of lung organoid in different groups after 72 h of challenge with H1N1-CA07 aerosols. The dead cells were stained by Propidium iodide (red), and the live cells were stained by Calcein (green).

The experiments were repeated three times independently with similar results.

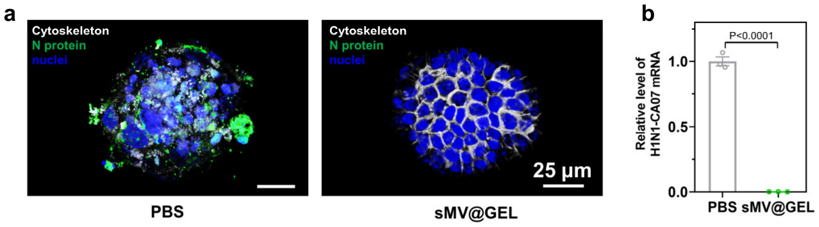

Fig. S35. The protection effect of sMV@GEL in child-derived HRT model.

(a) Representative 3D reconstructed CLSM images of lung organoids in different groups after 48 h of challenge with H1N1-CA07 aerosols. The cytoskeleton was stained by FITC-phalloidine (white, false color), cell nuclei were stained with DAPI (blue), and the N protein of H1N1-CA07 was immunofluorescently labeled with an N protein antibody and the corresponding secondary Alexa Fluor® 647 fluorescent antibody (green, false color).

(b) Relative level of H1N1-CA07 mRNA of lung organoids in different groups after 48 h of challenge with H1N1-CA07 aerosols (n=3 biologically independent experiments).

Data in b represent as means ± S.E.M. Statistical significance in b were calculated using two-tailed unpaired t-test. The P value of PBS to sMV@GEL was <0.0001. The experiments were repeated three times independently with similar results. Source data are provided in the Source data file.

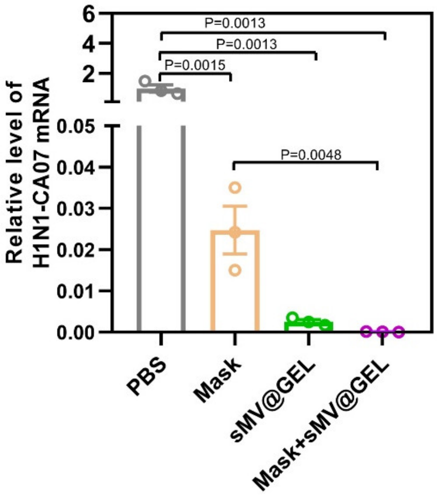

Fig. S36. Relative level of H1N1-CA07 mRNA of lung organoids in different groups after 48 h of challenge with high concentration H1N1-CA07 viral aerosols (n=3 biologically independent experiments). Four groups (PBS, mask, intranasal mask (sMV@GEL), combined masks (mask+sMV@GEL)) were included for comparative evaluation. To simulate real-life scenarios where masks may not be worn correctly, we introduced a "loose wearing" condition by creating a gap (1-3 mm) between the mask and the nasal module of HRT model. These data strengthened the applicability and effectiveness of the proposed intranasal mask as an additional protective measure against viral infections, particularly for healthcare workers and individuals in high-risk settings. Data represent as means ± S.E.M. Statistical significance was calculated using one-way ANOVA with multiple comparison tests. The experiments were repeated three times independently with similar results. Source data are provided in the Source data file.

Reference:

1. Tellier, R., Li, Y., Cowling, B.J. & Tang, J.W. Recognition of aerosol transmission of infectious agents: a commentary. *BMC Infect Dis* **19**, 101 (2019).
