# Supplementary material for: Intranasal mask for protecting the respiratory tract against viral aerosols
Source: Nat Commun. 2023 Dec 18;14:8398. doi: 10.1038/s41467-023-44134-w (PMC10728126; doi:10.1038/s41467-023-44134-w)
Supplement: Supplementary file 3 — Reporting Summary [file 41467_2023_44134_MOESM3_ESM.pdf]

nature portfolio

Corresponding author(s): Wei Wei, Guanghui Ma, Limin Wang

Last updated by author(s): Nov 20, 2023

Reporting Summary

Nature Portfolio wishes to improve the reproducibility of the work that we publish. This form provides structure for consistency and transparency in reporting. For further information on Nature Portfolio policies, see our Editorial Policies and the Editorial Policy Checklist.

Statistics

For all statistical analyses, confirm that the following items are present in the figure legend, table legend, main text, or Methods section.

n/a	Confirmed	
☐	☒	The exact sample size (*n*) for each experimental group/condition, given as a discrete number and unit of measurement
☐	☒	A statement on whether measurements were taken from distinct samples or whether the same sample was measured repeatedly
☐	☒	The statistical test(s) used AND whether they are one- or two-sided<br>*Only common tests should be described solely by name; describe more complex techniques in the Methods section.*
☒	☐	A description of all covariates tested
☐	☒	A description of any assumptions or corrections, such as tests of normality and adjustment for multiple comparisons
☐	☒	A full description of the statistical parameters including central tendency (e.g. means) or other basic estimates (e.g. regression coefficient) AND variation (e.g. standard deviation) or associated estimates of uncertainty (e.g. confidence intervals)
☐	☒	For null hypothesis testing, the test statistic (e.g. *F*, *t*, *r*) with confidence intervals, effect sizes, degrees of freedom and *P* value noted<br>*Give P values as exact values whenever suitable.*
☒	☐	For Bayesian analysis, information on the choice of priors and Markov chain Monte Carlo settings
☒	☐	For hierarchical and complex designs, identification of the appropriate level for tests and full reporting of outcomes
☒	☐	Estimates of effect sizes (e.g. Cohen's *d*, Pearson's *r*), indicating how they were calculated

Our web collection on statistics for biologists contains articles on many of the points above.

Software and code

Policy information about availability of computer code

Data collection	Images of aMV, sMV and SPV that were trapped by aMV were obtained using TEM (JEM-1400, JEOL). The size distribution of aMV or sMV were determined using Dynamic Light Scattering (ZetaSizer NANO ZS90, Malvern). Images of aMV@GEL was obtained using SEM (JEOL, JSM-7000F). Fluorescent images were collected using CLSM (A1/SIM/STORM, Nikon) or STED (SP8, Leica) with associated software. Flow-cytometry data were acquired via flow cytometry (CytoFLEX LX, Beckman Coulter). Images of SPV, aMV and aMV@GEL distribution were collected using an in vivo imaging system (530 FX Pro, Kodak) with associated software. Fluorescent data were obtained using imicroplate reader (Infinite M200, TECAN). The images of IHC and H&E staining were visualized by using an automatic multispectral imaging system (Vectra II, PerkinElmer). The gelation time of gel was detected by Rheometer (MCR302, Anton-Paar). The q-PCR data were obtained using the real-time PCR detection system (CFX96, Bio-Rad).Flow-cytometry data were acquired via flow cytometry (CytoFLEX LX, Beckman Coulter) with CytExpert software (version 2.3).
Data analysis	All statistical analyses were performed on GraphPad Prism (version 8.4.3). Flow-cytometry data were analyzed on CytExpert 2.3 .

For manuscripts utilizing custom algorithms or software that are central to the research but not yet described in published literature, software must be made available to editors and reviewers. We strongly encourage code deposition in a community repository (e.g. GitHub). See the Nature Portfolio guidelines for submitting code & software for further information.

Data

Policy information about availability of data

All manuscripts must include a data availability statement. This statement should provide the following information, where applicable:

- Accession codes, unique identifiers, or web links for publicly available datasets
- A description of any restrictions on data availability
- For clinical datasets or third party data, please ensure that the statement adheres to our policy

The main data supporting the results in this study are available within the paper and its Supplementary Information.
Source data for the figures in the main text and Supplementary Information are available at Figshare (https://doi.org/10.6084/m9.figshare.24590910.v1).
The code and cases for simulation in this study are available at GitHub (https://github.com/ScottFu123/simulation-files/tree/master).

Human research participants

Policy information about studies involving human research participants and Sex and Gender in Research.

Reporting on sex and gender	The CT data of human nasal cavity was derived from a volunteer (age 25, male).<br>Human lung organoids were derived from the surgery normal lung tissues adjacent to excised tumors obtained from a volunteer with advanced non-small cell lung carcinoma (age 63, female).
Population characteristics	The CT data of human nasal cavity was derived from a healthy, age 25, male Asian volunteer.<br>Human lung organoids were derived from the surgery normal lung tissues adjacent to excised tumors obtained from a volunteer with advanced non-small cell lung carcinoma (age 63, female, Asian).
Recruitment	The CT data was derived from one health volunteer with normal nasal cavity.<br>The normal lung tissues adjacent to excised tumors obtained from another volunteer.<br>All informed consent was obtained from the participants that providing the CT data of human nasal cavity or human lung tissue.
Ethics oversight	Human research involved in this study was approved and performed in accordance with the institutional guidelines of the Peking University First Hospital (Number: 2021-S-486), and informed consents were obtained from the participants (male volunteer who provided CT data of human nasal cavity and female volunteer who donated lung tissue).

Note that full information on the approval of the study protocol must also be provided in the manuscript.

Field-specific reporting

Please select the one below that is the best fit for your research. If you are not sure, read the appropriate sections before making your selection.

☒ Life sciences ☐ Behavioural & social sciences ☐ Ecological, evolutionary & environmental sciences

For a reference copy of the document with all sections, see nature.com/documents/nr-reporting-summary-flat.pdf

Life sciences study design

All studies must disclose on these points even when the disclosure is negative.

Sample size	In this study, sample size was determined by referring to pilot studies and relevant literature. We also referred to relevant literature to determine sample sizes. For in vitro and ex vivo experiments, the sample sizes was 3 biologically independent experiment. For the SPV inhibition experiments, the sample size was 3 biologically independent mice each group. For the H1N1 inhibition experiments, the sample size was 6 biologically independent mice each group.
Data exclusions	No data were excluded.
Replication	Experiments were replicated multiple times and obtained the similar results. For in vitro studies, experiments were replicated three times. For viral infection inhibition studies, experiments were replicated twice.
Randomization	For the in vitro experiments, samples were randomly allocated into experimental groups. For the in vivo studies, animals were randomly grouped.
Blinding	Investigators were not blinded for materials synthesis since the scientists must keep careful track of conditions. For the in vivo end-point evaluations, the investigators were blinded to group allocation during data collection and analysis.

Reporting for specific materials, systems and methods

We require information from authors about some types of materials, experimental systems and methods used in many studies. Here, indicate whether each material, system or method listed is relevant to your study. If you are not sure if a list item applies to your research, read the appropriate section before selecting a response.

Materials & experimental systems

n/a	Involved in the study
☐	☒ Antibodies
☐	☒ Eukaryotic cell lines
☒	☐ Palaeontology and archaeology
☐	☒ Animals and other organisms
☒	☐ Clinical data
☒	☐ Dual use research of concern

Methods

n/a	Involved in the study
☒	☐ ChIP-seq
☐	☒ Flow cytometry
☒	☐ MRI-based neuroimaging

Antibodies

Antibodies used

1. anti-human ACE2 (ab108252; Abcam; 1:100 dilution;clone: EPR4435(2))
2. anti-N protein of H1N1 (ab104870; Abcam; 1:100 dilution; clone: Polyclonal)
3. Goat Anti-Mouse IgG H&L (Alexa Fluor® 647)(ab150115; Abcam; 1:1000 dilution; clone: Polyclonal)
4. anti-human SCGB1A1 (DF3950; Affinity Biosciences; 1:100 dilution; clone: Polyclonal)
5. anti-human FOXJ1 (AF0372; Affinity Biosciences; 1:100 dilution; clone: Polyclonal)
6. anti-human P63 (DF6860;Affinity Biosciences; 1:100 dilution; clone: Polyclonal)

Validation

All antibodies were purchased from the supplier as noted above, and used without additional validation.
For antibodies 1: Dilutions were based on the manufacturer's (Abcam, https://www.abcam.cn) recommendations for flow cytometry.
For antibodies 1–3: Dilutions were based on the manufacturer's (Abcam, https://www.abcam.cn) recommendations for immunofluorescence.
For antibodies 2: Dilutions were based on the manufacturer's (Abcam, https://www.abcam.cn) recommendations for immunochemistry.
For antibodies 4–6: Dilutions were based on the manufacturer's (Affinity Biosciences, http://www.affbiotech.cn) recommendations for immunofluorescence.

Eukaryotic cell lines

Policy information about cell lines and Sex and Gender in Research

Cell line source(s)

293T cell (catalog no. CL-0005) and MDCK cell (catalog no. CL-0154) was purchased from Procell Life Science&Technology Co.,Ltd (Wuhan, China). Hnepc (catalog no. C303) was purchased from Honsun Biological Technology Co., Ltd (Shanghai, China).

Authentication

Cell line was authenticated by STR profiling.

Mycoplasma contamination

The cell line was tested for mycoplasma contamination. No mycoplasma contamination was found.

Commonly misidentified lines
(See ICLAC register)

No commonly misidentified cell lines were used.

Animals and other research organisms

Policy information about studies involving animals; ARRIVE guidelines recommended for reporting animal research, and Sex and Gender in Research

Laboratory animals

C57BL/6 mice and BALB/c mice(6–8 weeks) were purchased from Charles River (Beijing, China). Transgenic mice (6-8 weeks) with human ACE2 expression were purchased from GemPharmatech (Beijing, China).The mice were housed in an environmentally controlled room (23 °C, with 55 ± 5% humidity and under a 12 h–12 h light–dark cycle).

Wild animals

The study did not involve wild animals.

Reporting on sex

The study did not apply to only one sex.

Field-collected samples

The study did not involve samples collected from the field.

Ethics oversight

The mouse experiments were approved by the Institutional Animal Care and Use Committee at the Institute of Process Engineering,

Chinese Academy of Sciences (Number: IPEAECA2020042).

Note that full information on the approval of the study protocol must also be provided in the manuscript.

Flow Cytometry

Plots

Confirm that:

- [x] The axis labels state the marker and fluorochrome used (e.g. CD4-FITC).
- [x] The axis scales are clearly visible. Include numbers along axes only for bottom left plot of group (a 'group' is an analysis of identical markers).
- [x] All plots are contour plots with outliers or pseudocolor plots.
- [x] A numerical value for number of cells or percentage (with statistics) is provided.

Methodology

Sample preparation	ACE2-293T cells were collected, then filtered through a 40-µm cell strainer to isolate the cells for further analysis, as detailed in Methods.
Instrument	CytoFLEX LX flow cytometer (Beckman Coulter)
Software	Cyt-Expert software
Cell population abundance	At least 10000 cells were used for flow cytometric analysis.
Gating strategy	Cells were gated on the starting cell population in a FSC/SSC plot, followed by a FCA/FCH plot to gate single cells.

- [x] Tick this box to confirm that a figure exemplifying the gating strategy is provided in the Supplementary Information.
